# Early versus late surgical intervention for cervical spinal cord injury: A protocol for systematic review and meta-analysis

**DOI:** 10.1097/MD.0000000000033322

**Published:** 2023-03-24

**Authors:** Chaowei Yang, Xinming Yang

**Affiliations:** a Graduate School of Hebei North University, Zhangjiakou City, Hebei Province, China; b The First Affiliated Hospital of Hebei North University, Zhangjiakou City, Hebei Province, China.

**Keywords:** cervical spinal cord injury, decompression, meta-analysis, protocol, surgical timing

## Abstract

Acute traumatic cervical spinal cord injury (SCI) is a catastrophic event with substantial physical, emotional, and economic burdens to patients, families, and society. Spinal cord decompression is recommended for the treatment of acute SCI. However, the optimal surgical timing remains controversial. Therefore, we perform a protocol for systematic review and meta-analysis to compare the efficacy of early and late surgical intervention for acute SCI.

**Methods:**

This systematic review and meta-analysis follows the Preferred Reporting Items for Systematic Reviews and Meta-Analyses Protocols statement, which have been registered in advance in the International prospective register of systematic reviews (registration number: CRD42023397592). We will search the following databases for randomized controlled trials: the Cochrane Skin Group Trials Register, MEDLINE, EMBASE, the Cochrane Central Register of Controlled Trials, Chinese Biomedical Literature Database, Chinese Medical Current Content, and China National Knowledge Infrastructure. The risk of bias of the included studies will be appraised using the Cochrane Collaboration tool for randomized controlled trials. Statistical analysis will be performed using IBM SPSS Statistics (Armonk, NY).

**Result:**

The results of this systematic review will be published in a peer-reviewed journal.

**Conclusion:**

This systematic review will provide evidence regarding the optimal timing for spinal cord decompression in patients with acute SCI.

## 1. Introduction

Cervical spinal cord injury (SCI) causes several negative outcomes, including physical, mental, economical, and social impairments that affect not only the patient but also their family and caregivers.^[1–3]^ The number of patients is increasing yearly, and many studies regarding its treatment have been reported over the last several decades. Nevertheless, there is no recommended drug therapy for acute SCI, and stabilization and decompression are only recommended for acute SCI in guidelines published in 2013.^[4]^

There are 2 phases following acute cervical SCI: primary mechanical injury and secondary cellular injury. Primary mechanical injury is an irreversible damage caused by initial rapid spinal cord compression and trauma induced by a fracture or shearing force.^[5]^ Following primary injury, hemorrhage, vasospasm, ischemia, edema, excitotoxicity, inflammation, and apoptosis may bring about secondary cellular injury several hours after trauma, worsening the damage.^[6]^ The current management of SCI involves surgical intervention aimed at preventing and reducing secondary damage.

There are few large studies on the results of surgery in the early phase of cervical SCI. The results of previous cohort studies are conflicting. Some studies reported that early surgical intervention resulted in good neurological outcomes^[7]^; however, other studies have suggested that surgery in the early phase worsened neurological outcomes and survival rates.^[8]^

Current guidelines recommend surgical decompression within 24 hours of SCI, regardless of the level and severity of the injury.^[8]^ Nonetheless, in clinical practice, the decision to perform an emergent or urgent surgical decompression is still more often made based on the severity of the injury. This gap between guideline recommendations and clinical practice is likely due to the lack of high-quality evidence regarding the benefits of early surgical decompression. Given this, the optimal timing of surgical treatment remains unclear. Therefore, we perform a protocol for systematic review and meta-analysis to compare the efficacy of early and late surgical intervention for acute SCI.

## 2. Methods

This systematic review and meta-analysis follows the Preferred Reporting Items for Systematic Reviews and Meta-Analyses Protocols statement,^[9]^ which have been registered in advance in the International prospective register of systematic reviews (registration number: CRD42023397592). Ethical approval will not be required because this study is a systematic review.

### 2.1. Eligibility criteria

Inclusion criteria: patients with acute cervical SCI who received surgical decompression are eligible; the treatment group receives surgical decompression within 24 hours after admission, while the control group receives late surgical intervention (>24 hours); the primary outcome is the change of American Spinal Injury Association score from baseline to follow-up time after spinal injury. Secondary outcomes are neurological improvement rate, mortality, length of stay, and postoperative complications; the type of study is randomized controlled trial, and the language is limited to Chinese and English.

Exclusion criteria: repeatedly published papers; the literature is abstract, animal research, and cadaver study; the relevant data cannot be extracted from the published results, and the original data cannot be obtained after contacting the author.

### 2.2. Search strategy

We will search the following databases: the Cochrane Skin Group Trials Register, MEDLINE, EMBASE, the Cochrane Central Register of Controlled Trials, the Chinese Biomedical Literature Database, Chinese Medical Current Content, and China National Knowledge Infrastructure. Moreover, we will filter relevant medical journals and magazines to identify literature that is not included in the electronic databases. The preliminary search strategy for PubMed is presented in Table 1.

**Table 1:** Search strategy in PubMed.

Number	Search terms
#1	spinal cord injury [Ti/Ab]
#2	spinal cord contusion [Ti/Ab]
#3	spinal cord transection [Ti/Ab]
#4	traumatic central cord syndrome [Ti/Ab]
#5	#1 OR #2 OR #3 OR #4
#6	early surgical decompression [Ti/Ab]
#7	late surgical decompression [Ti/Ab]
#8	delay decompression [Ti/Ab]
#9	immediate decompression [Ti/Ab]
#10	early surgery [Ti/Ab]
#11	late surgery [Ti/Ab]
#12	#6 OR #7 OR #8 OR #9 OR #10 OR #11
#13	randomized [Ti/Ab]
#14	randomly [Ti/Ab]
#15	RCT [Ti/Ab]
#16	#13 OR #14 OR #15
#17	#5 AND #12 AND #16

### 2.3. Selection of studies

**Figure 1.**
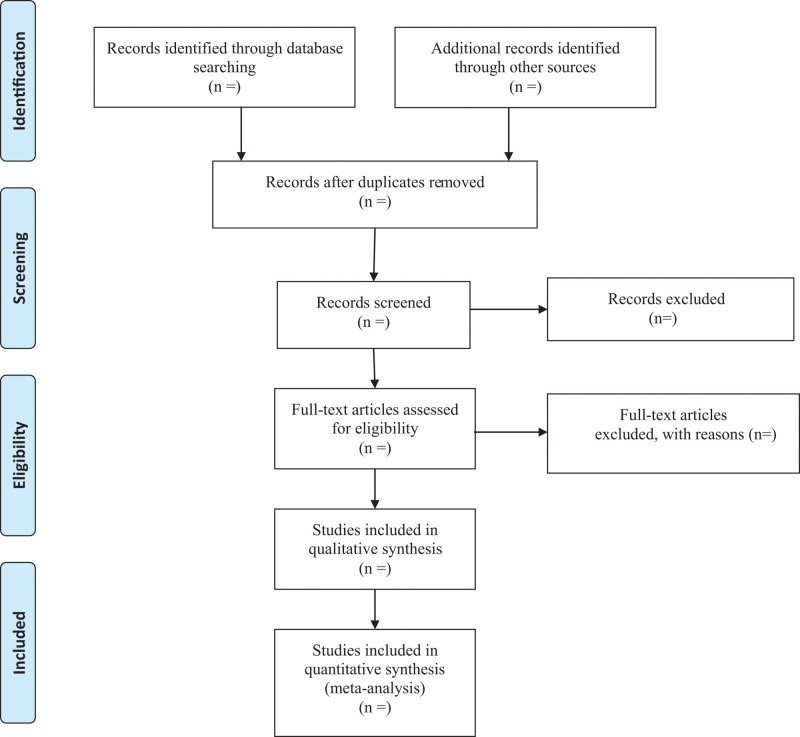
Flow diagram of included studies.

Note Express Version 3.2 (IBM) will be used for literature management. At first, duplicate documents will be deleted by the software. Then 2 reviewers will remove irrelevant articles independently by screening the titles and abstracts. If there is any uncertainty, we will obtain and read full texts. All the reasons for excluding studies will be recorded. Preferred reporting items for systematic reviews and meta-analysis flow chart has been drawn to illustrate the study selection procedure (Fig. 1).

### 2.4. Data extraction

Two reviewers will screen all the titles, abstracts, and full texts for inclusion, both of whom will be blinded to authors, journals, institutional affiliations, and dates of publication. Both reviewers will evaluate each selected reference independently and summarize relevant study characteristics. In the event of disagreement, a consensual decision between the 2 reviewers under the involvement of a third independent reviewer will be reached. The following data items will be extracted: the year of publication, study design, sample size, country of study, patient characteristics, time to surgery, and outcome measures. The corresponding authors of the original publications will be contacted via email in the event of insufficient data.

### 2.5. Risk of bias

The risk of bias of the included studies will be appraised using the Cochrane Collaboration tool for randomized controlled trials.^[10]^ The content of the evaluation involves sequence generation, allocation concealment, blinding of participants, blinding of outcome assessor, incomplete outcome data, reporting bias, and other bias. Each part of the evaluation contains a low risk of bias, a high risk of bias, or an unclear risk of bias.

### 2.6. Data synthesis and analysis

Statistical analysis will be performed using IBM SPSS Statistics (Armonk, NY). The risk ratio and 95% confidence intervals are collected for enumeration data, while the mean difference or standardized mean difference and 95% confidence intervals are used to calculate continuous outcome data. Heterogeneity will be detected by the *χ*^2^ test, and if the heterogeneity among studies is small (*P* > .05, *I*^2^ < 50%), a fixed-effects model analysis will be used; if the heterogeneity is high (*P* ≤ .05, *I*^2^ ≥ 50%), a random-effects model analysis will be used. Publication bias will be assessed using a funnel plot and the Begger test.

### 2.7. Sensitivity analysis

The sensitivity analyses will be performed by removing the studies with a high risk of bias in order to evaluate the stability of the results. To further confirm the stability of the above results, we exclude relevant studies sequentially for each outcome.

## 3. Discussion

Acute traumatic SCI is a catastrophic event with substantial physical, emotional, and economic burdens to patients, families, and society.^[11–13]^ The presentation of acute SCI can involve paralysis, numbness, or loss of bladder or bowel control.^[14]^ Despite investigative efforts into potential neuroprotective and regenerative therapies, there remain few treatment options for patients with acute SCI; for example, targeted blood pressure management, methylprednisolone, or spinal cord decompression.^[15–17]^ Urgent surgical decompression affords an early opportunity to restore spinal cord blood flow and mitigate secondary injury.^[18]^ There are strong data from experimental animal models to indicate that superior neurobehavioural outcomes are associated with early spinal cord decompression. Clinical data on this topic have been mixed as some studies have shown an effect of time of surgery whereas others have not, although there has been growing recognition that early decompressive surgery is a safe and reasonable treatment option. Differing time thresholds have been used to define early surgery after injury; of these, a cutoff of 24 to 48 hours has been studied most frequently. However, the role of decompression within 24 to 48 hours of acute SCI is controversial, and there is no definitive evidence of benefit. This systematic review will provide evidence regarding the optimal timing for spinal cord decompression in patients with acute SCI.

## Author contributions

**Conceptualization:** Chaowei Yang.

**Data curation:** Chaowei Yang.

**Investigation:** Xinming Yang.

**Methodology:** Xinming Yang.

**Writing – original draft:** Chaowei Yang.

**Writing – review & editing:** Xinming Yang.
